# In Vitro Study on Nematicidal Effect of Silver Nanoparticles Against *Meloidogyne incognita*

**DOI:** 10.3390/molecules30051132

**Published:** 2025-03-01

**Authors:** Ewa M. Furmanczyk, Dawid Kozacki, Wojciech Hyk, Magdalena Muszyńska, Malgorzata Sekrecka, Andrzej T. Skwiercz

**Affiliations:** 1 Department of Plant Protection, The National Institute of Horticultural Research, 96-100 Skierniewice, Poland; dawid.kozacki@inhort.pl (D.K.); malgorzata.sekrecka@inhort.pl (M.S.); andrzej.skwiercz@inhort.pl (A.T.S.); 2 Faculty of Chemistry, University of Warsaw, Pasteura 1, 02-093 Warsaw, Poland; wojhyk@chem.uw.edu.pl (W.H.); m.muszynska@chem.uw.edu.pl (M.M.); 3 Chemical Research Centre, University of Warsaw Biological, Żwirki i Wigury 101, 02-089 Warsaw, Poland

**Keywords:** root-knot nematode, nematicide, nematode management, nanoparticles, silver, zinc

## Abstract

Plant-parasitic nematodes remain a significant challenge to agriculture and horticulture. Unfortunately, there is a lack of rapid, efficient and eco-friendly control methods. Nanomaterials, therefore, appear to be a promising source of new plant protection strategies. In the current study, two nanosystems—based on one-component (Ag) or two-component (AgZn) colloidal systems—and an aqueous solution of AgNO_3_, were explored for their potential in nematode control, using *Meloidogyne incognita*—the most economically important root-knot nematode—as a model nematode. In vitro laboratory tests demonstrated high efficacy of all the tested compounds towards *M. incognita*. Incubation with an extremely low concentration of silver compounds (0.05 ppm) resulted in a 100% reduction of the *M. incognita* invasive larvae viability, as well as a 100% inhibition of the egg hatch process. Preliminary tests also showed no negative impact of tested compounds on seed germination. Thus, the nanosystems tested within this study offer a promising alternative to the current methods used for nematode control.

## 1. Introduction

Plant-parasitic nematodes (PPNs), with particular regard to root-knot nematodes (*Meloidogyne* spp.), pose a serious threat to agriculture and horticulture. In fact, the root-knot nematodes (RKNs) are recognized as the most harmful PPNs according to scientific and economic importance [1]. Global losses due to *Meloidogyne* infestations are estimated at USD 173 billion annually, across the main crops, such as wheat, tomatoes and potatoes [2]. The genus *Meloidogyne* consists of over 100 species [3] and is well known for its broad host range representing more than 3000 plant species, including economically important species such as cereals (e.g. *Triticum aestivum* L. or *Hordeum vulgare* L.), potato (*Solanum tuberosum* L.), tomato (*Solanum lycopersicum* L.), carrot (*Daucus carota* L.) and cucumber (*Cucumis sativus* L.) [4]. RKNs cause root galling, nutrient deficiencies and plant growth suppression, leading to yield loss [5]. *Meloidogyne* spp. are obligate sedentary endoparasites;almost all of their lifecycle takes place within the host plant. The life cycle starts with the egg and the subsequent three larval stages (J2-J4). Of these stages, J2 is capable of infecting the host plant and causing mechanical damage using the stylet. After entering the host plant, J2 starts to feed, which initiates the formation of giant cells. After three molts, J4 reaches the adult stage (male or female). Then the females lay eggs into gelatinous masses, which can be found on the surface of galled roots or embedded into gall tissue. One female may lay 500–2000 eggs under optimal conditions [6]. Therefore, only the egg and the J2 larvae stages can be targeted for effective plant protection against *Meloidogyne*. However, the high reproduction rate and the polyphagous lifestyle, combined with the presence of a cuticle acting as a highly impervious barrier between the animal and its environment, make plant protection against *Meloidogyne* a difficult and challenging task.

Chemical control with synthetic nematicides is still the most effective strategy to control nematodes. However, many of them are broad spectrum pesticides that are active against not only nematodes but also insects, bacteria and fungi, causing a negative environmental impact, especially to the soil [7]. This is not in line with the current trends in promoting environmental biodiversity and reducing the use of pesticides, such as the Green Deal and Farm to Fork strategies included within the “EU soil strategy for 2030” [8]. More eco-friendly methods of PPN control include soil management practices, such as crop rotation, trapping with suppressive plants or application of organic amendments. However, the effectiveness of these practices, as well as the usage of microorganisms (including strains of *Bacillus*, *Pseudomonas*, *Metarhizium*, *Purpureocillium*, *Steinernema*, etc.), is influenced by environmental conditions—especially temperature and humidity—aside from any individual soil physical-chemical characteristics [9].

Nanomaterials are emerging as promising tools in agriculture, with silver nanoparticles standing out due to their potent antimicrobial properties and potential selective toxicity against certain pathogens. They could be used as nano-pesticides, nano-fertilizers or pesticide nano-carrier agents, thus reducing the doses of broad-spectrum pesticides used and enables the rational management of water resources [10,11,12]. There are few reports showing the nematicidal properties of silver nanoparticles, also against the J2 larvae of *M. graminicola* [13], *M.incognita* [14] or other PPN species like: *Xiphinema diversicaudatum*, *Ditylenchus dipsaci* or *Heterodera schachtii* [15].

In this study, we present the effects of different silver species, including silver, silver and zinc nanoparticle systems (AgNPs, AgZnNPs) and silver cations (AgNO_3_), on the motility, mortality and hatching rate of the *M. incognita* J2 and egg stages as a model species of the genus *Meloidogyne*. A comprehensive evaluation of the effects on both agriculturally important stages is crucial for the selection of the concentrations of silver compounds for further development of integrated plant protection against root-knot nematodes.

The aqueous systems of nanoparticles employed in this study were synthesized according to our recently designed synthetic method [16]. In contrast to commercially available nanoparticle formulations, our method does not require the addition of any chemical/biological initiators, external stabilizing agents or supporting electrolytes. The nanoparticle systems are prepared in ultrapure water and are stabilized electrostatically by the ionic species that make up the particles. The absence of other substances (additives) in the synthesized materials significantly simplifies the chemical matrix of the systems. This unique feature allows the nanoparticle aqueous solutions to be considered matrix-less systems.

## 2. Results

### 2.1. Effects of Silver Compounds on Juvenile Mortality

All three examined compounds showed nematicidal potential towards the *M. incognita* juveniles tested in vitro conditions (Table 1). All tested compounds in each tested concentration showed significantly changed nematode mobility when compared to the appropriate control. However, the effect was dependent on the concentration used. The three highest concentrations tested (0.05 ppm, 0.10 ppm and 0.50 ppm) showed 100% immobilization of the *M. incognita* J2 larvae. The 0.01 ppm concentrations of tested compounds resulted in the lowest (50–79%) immovability of J2 larvae, while 0.02 ppm manifested almost full (92–99%) immobilization of *M. incognita* individuals. The greatest changes in *M. incognita* mobility occurred during the first 24 h of incubation. The nonmotile share recorded thereafter (48 h and 72 h after addition of tested compounds) remained very similar to the effect observed after 24 h of incubation.

**Figure 1:**
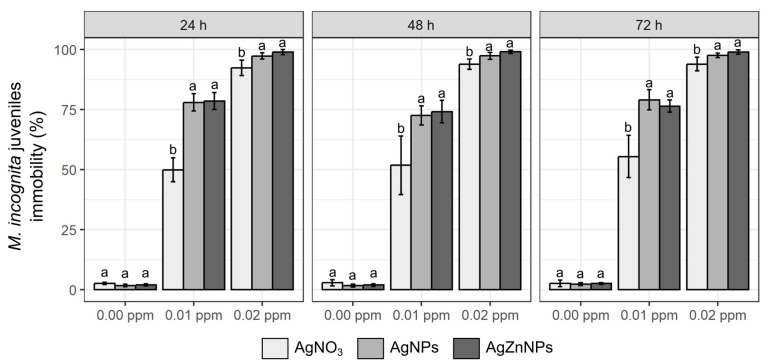
Percentage of immobility of *M. incognita* juveniles after incubation with different silver compounds in laboratory tests. The results are presented as means (n = 4) ±SD. Different letter shows statistically significant differences between all tested compounds, at the same concentration, after certain incubation time for *p* ≤ 0.05.

When we compared the immobility rate resulting from the use of different compounds at the same concentration and after the same incubation time, we also found statistically significant differences (Figure 1). The AgNO_3_ 0.01 ppm treatment resulted in the immobilization of around 50% of the J2 population, while the AgNPs and AgZnNPs 0.01 ppm treatments showed higher immobilization rates (72–79%) for both nanoparticle compounds. Significant differences between the silver nitrate and nanosilver compounds were also observed for 0.02 ppm treatments, where the nanoparticles showed almost complete immobilization of J2 (97–9%) and the AgNO_3_ treatment resulted in slightly lower but significant immobilization of J2 larvae (92–94%). Treatments with higher concentrations of tested compounds (0.05–0.50 ppm) resulted in 100% immobility of *M. incognita* J2 larvae for all three tested compounds (Table 1). However, the effects obtained for both nanoparticles’ formulations of silver always gave comparable results, without significant differences between AgNPs and AgZnNPs.

**Figure 2:**
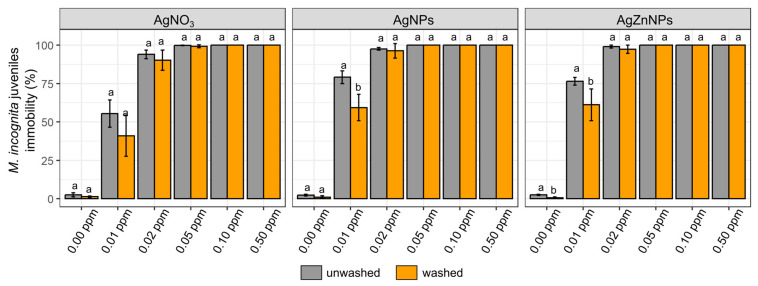
Comparison of percentage of immobility of *M. incognita* juveniles after 72 h of incubation with selected silver compounds before and after washing procedure. Different letter shows statistically significant differences between each tested compound at the same concentration before and after washing-up procedure for *p* ≤ 0.05.

To assess if the observed effect was only temporary and could be reversed, we transferred the *M. incognita* juveniles, after 72 h of incubation with appropriate compounds, into clean miliQ water and incubated for another two hours. Then the number of dead nematodes was determined once again and compared to the results obtained after 72 h of incubation (Figure 2).

Statistical analysis of the results showed significant differences between washed and unwashed *M. incognita* juveniles’ viability after 72 h exposure to the lowest tested concentrations of AgNPs and AgZnNPs. However, the effect observed at higher concentrations of nanoparticles used was stable and showed no differences between washed and unwashed *M. incognita* communities. The washing process has no effect on the *M. incognita* juveniles’ mobility recorded after incubation with silver nitrate, although this may also be the result of relatively high variations between replicates.

### 2.2. Effects of Silver Compounds on Eggs Hatching

**Figure 3:**
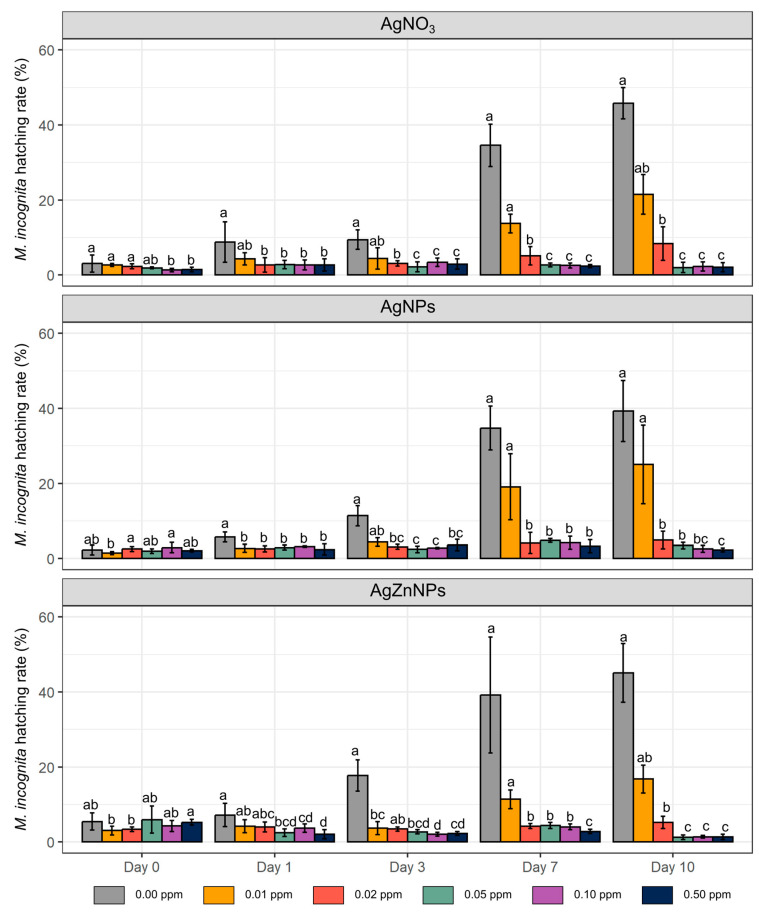
Percentage of hatching of *M. incognita* eggs in laboratory tests. The results are presented as means (n = 4) ±SD. Different letter shows statistically significant differences between different timepoints and compound concentration within one specific compound (AgNO_3_ or AgNPs or AgZnNPs) based on the Kruskal–Wallis test with Benjamini–Hochberg correction (*p* ≤ 0.05).

After the first day of the incubation, a visible and statistically significantly lower hatching rate (percentage of J2 larvae of the total number of eggs at the beginning of the experiment, without differentiating motile or nonmotile individuals) was observed for 0.02 ppm and higher concentrations for AgNO_3_ and AgNPs, while for AgZnNPs, a similar effect was observed only for 0.05 ppm and higher concentrations (Figure 3). These trends were then stable for the whole experiment. Also, 0.02 ppm AgZnNPs treatment significantly lowered the hatching abundance of *M. incognita* eggs, but after the longer incubation time (7 days or longer). The minor differences in the number of larvae observed at the start of the experiment (Day 0) are probably the result of the unsynchronized nematode inoculum used for the experiment.

**Figure 4:**
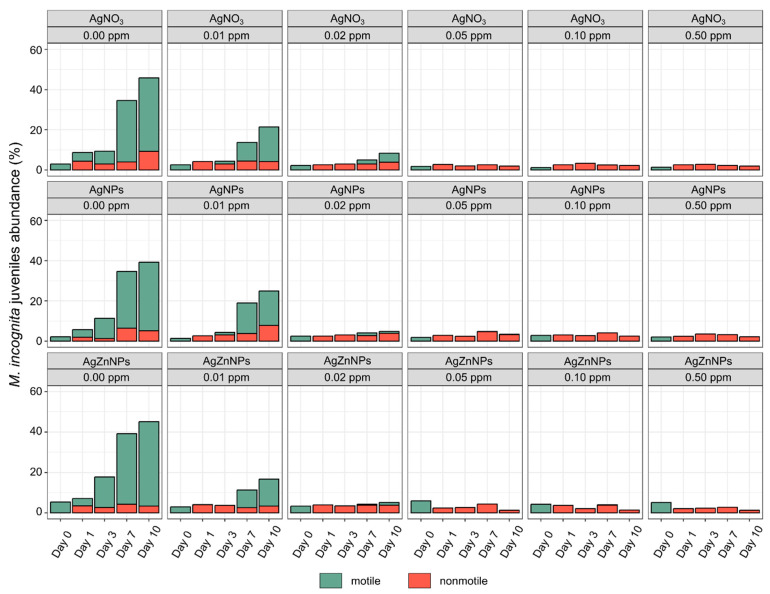
Percentage share of motile and nonmotile hatched *M. incognita* juveniles after incubation for different times and different concentrations of tested silver compounds. The results are presented as means (n = 4). Detailed statistical analysis of the nonmotile individuals’ abundance is present in Table S1.

The comparison of the abundance of motile and nonmotile *M. incognita* juveniles, hatched after the incubation with different concentrations of three tested compounds (Figure 4), showed that regardless of the compound used, motile juveniles could be observed only in two lower concentrations (0.01 ppm and 0.02 ppm). In other words, in this range of concentrations, *M. incognita* was able both to hatch from the egg and then survive in this environment. Higher concentrations of AgNO_3_, AgNPs, AgZnNPs seemed to almost completely inhibit hatching. Moreover, any individual larvae that were able to hatch were observed as nonmotile (Figure 4). The statistical analysis of the percentage of nonmotile larvae, observed from Day 1 to Day 10 of the experiment with different compounds, showed some small differences between tested compounds (Table S1). In most cases, a stronger potential effect of AgNPs and AgZnNPs in comparison to AgNO_3_ (0.05 ppm after 7 days of incubation) or of AgNPs in comparison to AgZnNPs (0.05 ppm after 10 days of incubation) was shown. At the end of the experiment (Day 10), ten randomly selected eggs from treatments with 0.02 ppm or 0.05 ppm concentrations (as the closest concentrations to the threshold that resulted in total inhibition of hatching) were rinsed with miliQ and transferred to a new plate incubated in miliQ water for a subsequent 10 days to determine whether the effect of the compounds used was reversible. No hatching was observed.

### 2.3. Effect of Silver Compounds on Seed Germination

Radish seed germination tests were carried out at two selected concentrations for each silver compound. A concentration of 0.05 ppm was chosen as the lowest concentration, at which 100% larval immobility and almost no hatching of *M. incognita* eggs was observed. The concentration of 0.50 ppm was chosen as the highest concentration tested for *M. incognita* experiments. No significant differences were observed for the percentage of germinated seeds after 7 days of incubation with different silver compounds, in comparison to the control (Table 2).

## 3. Discussion

Our results showed 100% immobility of J2 larvae (which could be considered as 100% mortality according to the results of our experiments) and a complete absence of transition from the egg stage to the viable J2 invasive stage after just 24 h of incubation with all tested silver compounds at a concentration of 0.05 ppm. A significantly higher toxicity of silver nanoparticles to *M. inocgnita* J2 larvae, in comparison to the ionic form (AgNO_3_ treatment), was only demonstrated at the lowest concentrations tested (0.01–0.02 ppm). Silver nanoparticles with large surface areas can provide better contact for interaction than the ionic form, thus being considered more toxic [17]. Moreover, the nanoparticles studied here constitute a two-component colloidal system (ionic and nanoparticle forms), so we could also observe the combined effect of the Ag^+^ and AgNPs. Nevertheless, all silver compounds tested within this work seemed to be highly toxic to the *M. incognita* as no significant differences between them were observed at higher concentrations. There are several studies reporting the nematicidal potential of silver nanoparticles or green synthesized silver nanoparticles mediated by various plant species extracts towards *M. incognita* or other *Meloidogyne* spp. However, in comparison to our research, they usually tested higher concentrations of the nanoparticles. Elarabi et al. [18] tested two different kinds of nanoparticles (Ag or ZnO nanoparticles) in the range between 100 ppm and 300 ppm, and showed 100% mortality of J2 larvae in the highest nanoparticles concentration. Nazir et al. [19] investigated the effect of 25–100 ppm of silver nanoparticleson the egg hatchability and survival of J2 *M. incognita* larvae, showing 100% mortality only at the highest concentrations tested. Cromwell et al. [20] tested silver nanoparticles, in the range of up to 150 ppm, towards J2 *M. incognita* larvae and, after 6 h of exposure to 30–150 ppm Ag nanoparticles, recorded more than 99% of inactive nematodes. Baronia et al. [13] tested the lowest concentration of silver nanoparticles (0.01–50 ppm) towards J2 larvae of *M. graminicola* and identified 0.10 ppm as the lowest concentration for 100% mortality after 12 h of incubation. Moreover, Fouda et al. [21] tested the microcrystalline cellulose embedded silver nanoparticles (0.01–50 ppm) towards J2 larvae of *M. incognita* and showed more than 95% mortality in a 40 ppm treatment after 72 h of incubation. Other researchers tested green silver nanoparticles, which also contained plant based compounds derived from species, such as *Glycyrrhiza glabra* [22], *Ficus sycomorus* [23], *Cassia fistula* [24] or even supernatant from *Photorhabdus luminescens* culture—a bacterium symbiotic with the entomopathogenic nematode, *Heterorhabditis indica* [25]. Nevertheless, nanoparticle concentrations causing 100% J2 larval mortality or 100% inhibition of *M. incognita* egg hatching were 0.5 ppm in the most effective cases where nanoparticles synthesized with a *G. glabra* aqueous root extract were used [22]. The differences in toxicity of the individual nanoparticle preparations reported above may result from differences in the size and shape of the nanoparticles [17], or the different sensitivity of the *Meloidogyne* isolates used in the experiments, similar to the varying sensitivity of divergent mutants of *Caenorhabditis elegans* to silver nanoparticles [26].

Unfortunately, the precise mechanism of action of nanoparticles on nematodes is not well understood. However, it seems to be similar to the mode of action of nanoparticles proposed for microorganisms. The cuticles of nematodes, like the membranes of bacteria, are negatively charged [27], attracting positively charged silver nanoparticles which can interact directly with the cell membrane, leading to an increase in its permeability and destruction [28]. Moreover, nanoparticles can enter a nematode body through the pharynx and the vulva, as it was observed for the free living nematode *C. elegans* and for silver nanoparticles [26]. Thus, in the case of more complex organisms like nematodes, nanoparticles can cause a toxic effect by interacting directly with the internal tissues. Interestingly, the 24 h exposure of *C. elegans* to 0.1 ppm silver nanoparticles did not affect the nematode survival; although, it resulted in a significant upregulation of genes associated with nuclear signalling and a downregulation of genes involved in stimulus, locomotion, feeding and reproductive behaviour [26]. Other studies on the effects of silica nanoparticles on *C. elegans* showed their localization in the cytoplasm and cell nucleus, where they induced an accumulation of insoluble ubiquitinated proteins and nuclear amyloid leading to premature ageing of the nematode [29]. All of these findings are then consistent with mechanisms proposed for microorganisms, including the production of a reactive oxygen species inducing stress conditions, that can lead to nucleic acid damage, protein oxidation and degradation and finally disruption of cell structure and function [28]. On the other hand, studies on mammalian cells showed that AgNPs and Ag^+^ could operate through different pathways in cell damage; AgNPs make cell death through lipid peroxidation leading to proteotoxicity, while cationic species of Ag generate H_2_O_2_, which significantly increases oxidative stress [30]. A promising method, that can better understand the mechanism of the action of silver nanoparticles, is the advanced luminescent approach to detect proteins by living cell labeling with nanoparticles [31].

According to our preliminary experiments, the tested silver compounds appeared to be safe for plants in the concentration range tested; they showed no effect on radish seed germination, in contrast to other nanoparticles [32]. However, future research should also focus on the interactions between nanoparticles and the environment, in particular, the effect on the soil microbiome, which can now be easily assessed by NGS [33,34] and the feedback from natural conditions (temperature, humidity, soil physicochemical properties or exposure to sunlight) that shape the stability and mobility of nanoparticles in the environment [35,36]. Indeed, such research can help to determine the precise dose and application methods of nanoparticles, and is therefore an essential part of the cost-effectiveness analysis of the suggested solution. Nevertheless, in the absence of both effective and environmentally friendly nematode control options in agriculture and horticulture, further research on nanoparticles is desirable and needed. There are two potential advantages of using our customized colloidal systems for testing their nematicidal effect. Firstly, because of their matrix-less nature, their application eliminates uncontrolled accumulation of other substances in the environment. Such substances, delivered along with active nanoparticles during plant treatments, are usually potential sources of contamination and/or process inhibitors. Secondly, the presence of both ionic and nanoparticle forms of a metal in the colloid system may enhance its biochemical activity, making the nanosystem a more effective agent at a lower total concentration of the metal; this is supported by our observations for the 0.01–0.02 ppm concentrations tested in experiments with J2 invasive larvae.

## 4. Materials and Methods

### 4.1. Characteristics of NPs Used in the Study

Two nanosystems, namely Ag and Zn (in the final form of ZnO) were synthesized in aqueous solutions according to our recently developed method [16]. The synthesis of one-component nanoparticle systems of Ag and Zn employed the anodic dissolution of high-purity electrode material (made of either silver or zinc) immersed in ultrapure water under the conditions of variable potential. The variable potential polarization of the electrodes resulted in the generation of hydrated metal cations which were transported (by diffusion and migration) to the counter electrode (made of the same metal). At the counter electrode the cationic species were reduced to a metallic form and nucleated to form a nanoparticle. The Ag and Zn nanosystems were used as the bases for one-component (Ag) and two-component (AgZn) colloidal systems. The latter formulations were prepared by mixing the Ag preparation with ZnO colloidal systems. All preparations were analyzed using SP-ICP-MS. A summary of the most important parameters is provided in Table 3. The aqueous solution of AgNO_3_ was prepared by dissolving solid AgNO_3_ salt (Pol-Aura, Poland, 99%) in ultrapure water to obtain the Ag+ concentration identical to the total Ag concentration in the AgNPs system.

### 4.2. Source of M. incognita

The culture of root-knot nematode, *Meloidogyne incognita* (Chitwood, 1949), was maintained on cucumber (*Cucumis sativus* L.) roots for 60 days. Eggs were obtained from infested roots of cucumber by 0.5% sodium hypochlorite solution followed by shaking for 2 min. Juveniles J2s were then gathered by hatching methodology for approximately ten days [37].

### 4.3. Effects of Silver Compounds on Juvenile Immobility and Mortality

The effect of silver compounds was assessed in vitro using 24-well plates. To each well approximately 200–250 of *M. incognita* juveniles were added in 750 ul od miliQ water. Then, an equal volume of two times concentrated solutions of appropriate compounds was added to obtain final concentrations, as follows: 0.00 ppm, 0.01 ppm, 0.02 ppm, 0.05 ppm, 0.10 ppm and 0.50 ppm. The experiment was conducted in four replicates. The plates were incubated at 28 °C. The mortality of juveniles was recorded after 24, 48 and 72 h. Nematodes were considered alive if they moved or had a wriggling shape, and dead if they took a straight shape and did not move. After 72 h of incubation, the contents of each well were rinsed twice with 2 mL miliQ water. After suspension in another 2 mL miliQ water, they were incubated for 2 h and then finally mortality was assessed.

### 4.4. Effects of Silver Compounds on Eggs Hatching

The effect of silver compounds was assessed in vitro using 24-well plates. To each well, approximately 100–200 *M. incognita* eggs were added in750 µL of miliQ water. Then an equal volume of two times concentrated solutions of appropriate compounds was added to obtain final concentrations, as follows: 0.00 ppm, 0.01 ppm, 0.02 ppm, 0.05 ppm, 0.10 ppm and 0.50 ppm. The experiment was conducted in four replicates. The plates were incubated at 28 °C. The number of hatched eggs and the viability of the hatched juveniles was recorded on days 1, 3, 7, 10 and 14. Hatched juveniles were considered alive if they moved or had a wriggling shape, and dead if they took a straight shape and did not move. The percentage of the total number of motile and nonmotile juveniles in comparison to the total number of eggs on day 0 was used to calculated the hatching rate.

After 10 days of incubation, 10 eggs from each replicate of 0.00 ppm, 0.02 ppm and 0.05 ppm concentrations of each nanoparticle solution were transferred to a new plate using automatic pipet, rinsed and resuspended in miliQ water. An additional 10 days of observation of eggs hatching was performed to assess if the effect of nanoparticles could be reversible.

### 4.5. Effect of Silver Compound on Seed Germination

Seeds of red radish (*Raphanus sativus* L. var. Carmen) were obtained from a commercial source (P.P.H.U. “Ogrodnik”, Poland). Seeds were disinfected with sodium hypochlorite (3%) for 5 min, washed thoroughly with miliQ water and then germinated in Petri dishes (50 seeds per plate) on filter paper moistened with miliQ water or with one of three silver compound solutions (AgNO_3_, AgNPs or AgZnNPs) in 0.05 ppm or 0.50 ppm concentration. The germination was carried out in the dark at 23 °C. The number of germinated seeds was recorded after 7 days of incubation. The experiment was set up in four replicates.

### 4.6. Statistical Analysis

Statistical analysis of data was performed using the R software version 4.1.3 [38]. The Shapiro-Wilk test was used to verify if the data followed a normal distribution and the Levene’s test was used to verify the homogeneity of variances. The data were thus analyzed by ANOVA and means differences were tested with Tukey’s test at *p*  ≤  0.05 with HSD test function from the “agricolae” package. In case of abnormal distribution, the non-parametric Kruskal-Wallis analysis with Fisher’s least significant difference post hoc test was utilized, introducing the Benjamini-Hochberg correction, with significance set at *p*  ≤  0.05, using the Kruskal function from the “agricolae” package.

## 5. Conclusions

The nanoparticles presented and described in this paper offer a promising alternative to methods used for plant protection against nematodes. Traditional practices are based on the use of intercropping or crop rotation, which are time-consuming and do not allow a rapid response to the nematode threat present in the environment. Chemical plant protection products with nematicidal properties, on the other hand, are usually harmful to other living organisms. Thus, our preliminary tests showing the high efficiency of nanoparticles and silver nitrate at very low concentrations and the lack of negative effects on seed germination are very promising. However, further research is needed to determine the environmental impact of nanoparticles and their effectiveness against nematodes under greenhouse or in open field conditions.

## Figures and Tables

**Table 1:** Percentage of immobility of *M. incognita* juveniles in laboratory tests. The results are presented as means (n = 4) ±SD. Different letter shows statistically significant differences between different timepoints and compound concentration within one specific compound (AgNO_3_ or AgNPs or AgZnNPs), based on the Kruskal–Wallis test with Benjamini–Hochberg correction (*p* ≤ 0.05).

Incubation Period	AgNO_3_					
	0.00 ppm	0.01 ppm	0.02 ppm	0.05 ppm	0.10 ppm	0.50 ppm
24 h	2.6 ± 0.5 e	49.9 ± 5 d	92.4 ± 3.3 c	100.0 ± 0.0 a	100.0 ± 0.0 a	100.0 ± 0.0 a
48 h	2.9 ± 1.3 e	51.8 ± 12.2 d	93.9 ± 2.2 c	100.0 ± 0.0 a	100.0 ± 0.0 a	100.0 ± 0.0 a
72 h	2.6 ± 1.4 e	55.4 ± 8.8 d	93.9 ± 2.8 c	99.8 ± 0.2 b	100.0 ± 0.0 a	100.0 ± 0.0 a
**Incubation period**	**AgNPs**					
	**0.00 ppm**	**0.01 ppm**	**0.02 ppm**	**0.05 ppm**	**0.10 ppm**	**0.50 ppm**
24 h	1.7 ± 0.5 e	78 ± 3.6 cd	97.4 ± 1.3 b	100.0 ± 0.0 a	100.0 ± 0.0 a	100.0 ± 0.0 a
48 h	1.7 ± 0.6 e	72.6 ± 4 d	97.4 ± 1.4 b	100.0 ± 0.0 a	100.0 ± 0.0 a	100.0 ± 0.0 a
72 h	2.4 ± 0.6 e	79.1 ± 4.2 c	97.6 ± 0.9 b	100.0 ± 0.0 a	100.0 ± 0.0 a	100.0 ± 0.0 a
**Incubation period**	**AgZnNPs**					
	**0.00 ppm**	**0.01 ppm**	**0.02 ppm**	**0.05 ppm**	**0.10 ppm**	**0.50 ppm**
24 h	2.0 ± 0.5 d	78.6 ± 3.6 c	98.8 ± 0.9 b	100.0 ± 0.0 a	100.0 ± 0.0 a	100.0 ± 0.0 a
48 h	2.0 ± 0.5 d	74.2 ± 4.7 c	99.1 ± 0.7 b	100.0 ± 0.0 a	100.0 ± 0.0 a	100.0 ± 0.0 a
72 h	2.6 ± 0.4 d	76.5 ± 2.5 c	99.0 ± 0.9 b	100.0 ± 0.0 a	100.0 ± 0.0 a	100.0 ± 0.0 a

**Table 2:** Percentage of germinated seeds after incubation with the tested compounds. The same letter stands for the statistically insignificant differences between treatments for *p* ≤ 0.05. The results are presented as means ± SD (n = 4).

miliQ Water	AgNO_3_		AgNPs		AgZnNPs	
	0.05 ppm	0.50 ppm	0.05 ppm	0.50 ppm	0.05 ppm	0.50 ppm
47.0 ± 5.3 a	50.0 ± 3.3 a	45.5 ± 7.0 a	49.5 ± 3.0 a	51.5 ± 6.4 a	50.0 ± 5.2 a	48.0 ± 9.9 a

**Table 3:** Summary of physicochemical characteristics of nanoparticle (NP) systems employed in this work. The quantification results are associated with an expanded uncertainty at the 95% confidence level (number of replicated measurements = 5).

Type of NP Preparation	Analyte	NP Size		Number of NPs (× 10^5^)	Concentration of Metal		Total Metal Content (mg/L)
		Mean Size (nm)	Most Frequent Size (nm)		in NP Form (µg/L)	in Ionic Form (mg/L)	
Ag	Ag	52 ± 1.15	44 ± 0.88	241 ± 42	12.92 ± 0.68	10.84 ± 0.3	10.85 ± 0.3
AgZn	Ag	42 ± 2.09	35 ± 1.09	603 ± 116	13.6 ± 1.17	4.71 ± 0.06	4.72 ± 0.07
	Zn	69 ± 1.02	67 ± 1.04	5 ± 0.55	0.5 ± 0.04	1.21 ± 0.02	1.22 ± 0.013

## Data Availability

Dataset available on request from the authors.

## Supplementary Materials

The following supporting information can be downloaded at: https://www.mdpi.com/article/10.3390/molecules30051132/s1, Table S1: Hatching rate of *M. incognita* eggs in laboratory tests. The results are presented as means (n = 4) ±SD. Different letter showed statistically significant differences within the same row tested based on ANOVA and means differences tested with Tukey’s test or the Kruskal–Wallis test with Benjamini–Hochberg correction (*p* ≤ 0.05)—rows marked with asterix (*).
